# Research progress of biomass carbon materials as anode materials for potassium-ion batteries

**DOI:** 10.3389/fchem.2023.1162909

**Published:** 2023-04-27

**Authors:** Xiang Li, Yimiao Zhou, Bin Deng, Jiani Li, Zuowei Xiao

**Affiliations:** ^1^ College of Pharmacy, Hunan University of Chinese Medicine, Changsha, China; ^2^ Key Disciplines of Pharmacy, Hunan University of Chinese Medicine, Changsha, Hunan, China; ^3^ Hunan University of Chinese Medicine and Food Homology Innovation Laboratory, Changsha, Hunan, China; ^4^ Xiangxing College of Hunan University of Chinese Medicin (Xiangyin Campus), Changsha, China; ^5^ School of Food Science and Engineering, South China University of Technology, Guangzhou, China

**Keywords:** potassium-ion battery, biomass carbon, anode materials, doping, storage capacity

## Abstract

Biochar materials have attracted people’s attention because of their environmental friendliness, abundant resources, and the use of waste resources for reuse. As a potassium-ion anode material, biomass char materials synthesized by different methods have broad application prospects. However, due to the problems of low initial magnification and limited potassium-storage capacity, it is necessary to improve the electrochemical performance through modifications, such as atomic doping. Atomic doping is an effective way to improve battery conductivity and potassium storage. In this paper, the synthesis method of biochar as an anode material for potassium-ion batteries and the influence of atomic doping on its modification in recent years are reviewed.

## 1 Introduction

Lithium and potassium atoms belong to the same major group and have similar physicochemical characteristics. Both lithium-ion and potassium-ion batteries operate on a similar concept. The K + -rich electrolyte solution is used to transport potassium ions between the cathode and the anode during the charge/discharge process (Manthiram 2017; Li et al., 2021; Zhou et al., 2021). Therefore, the storage capacity of potassium ions is one of the key factors to promote the rapid development of potassium-ion batteries; although due to the wide radius of potassium ions, its capacity is limited. Investigation of anode materials for potassium-ion batteries with superior electrochemical properties is currently one of the key research areas (Sha et al., 2020; Nadeem et al., 2022).

The anode components of potassium-ion batteries can be divided into alloy components, carbon-based components, titanium-based components, conversion components, and organic components (Zhang et al., 2021). Carbon-based materials are good at reversibly removing or embedding potassium as a negative electrode material. They also offer the benefits of environmental protection, non-toxicity, and stable performance. Graphite and non-graphite electrode materials are the two types of carbon-based materials. Graphite and graphene are ascribed to graphite-based electrode materials, while soft and hard carbon are attributed to non-graphite-based electrode materials. Amorphous carbon, a unique substance having a disordered structure and an ordered carbon layer, includes soft carbon as a subtype. High levels of graphitization are challenging to obtain hard carbon. There are many holes and disordered structures in carbon-based materials that are composed of microcrystalline and amorphous areas of graphite. Because it is abundant in resources, ecologically benign, and capable of reusing waste materials, hard carbon has attracted people’s interest (Izanzar et al., 2018; Yuan et al., 2022). Raw material sources can be divided into plant, animal, and microbial carbon materials. Plant biochar materials include hemp straw (Wang et al., 2021b), willow leaves (Qu et al., 2015), pine nut shells (Wang et al., 2019), and walnut shells (Zhang et al., 2021). Animal-based biochar includes chicken bones (Yuan et al., 2021), and microbial carbon materials include *Ganoderma lucidum* spore powder (Yang et al., 2020).

Although hard carbon materials have been widely used as anode materials for potassium-ion batteries, their rate performance and cycle stability are poor. Moreover, the limited interlayer spacing prevents the insertion of larger potassium ions (Pei et al., 2020; Gao et al., 2021) It needs to be modified to have a better performance. It is found that heteroatom doping can effectively improve the speed and energy density of ion and electron transport and then improve the rate performance of hard carbon materials (Liu et al., 2019; Ma et al., 2021). In this paper, the research progress of biomass carbon materials used as anode materials for potassium-ion batteries in heteroatom doping is reviewed.

## 2 Synthesis of biomass carbon materials

**FIGURE 1:**
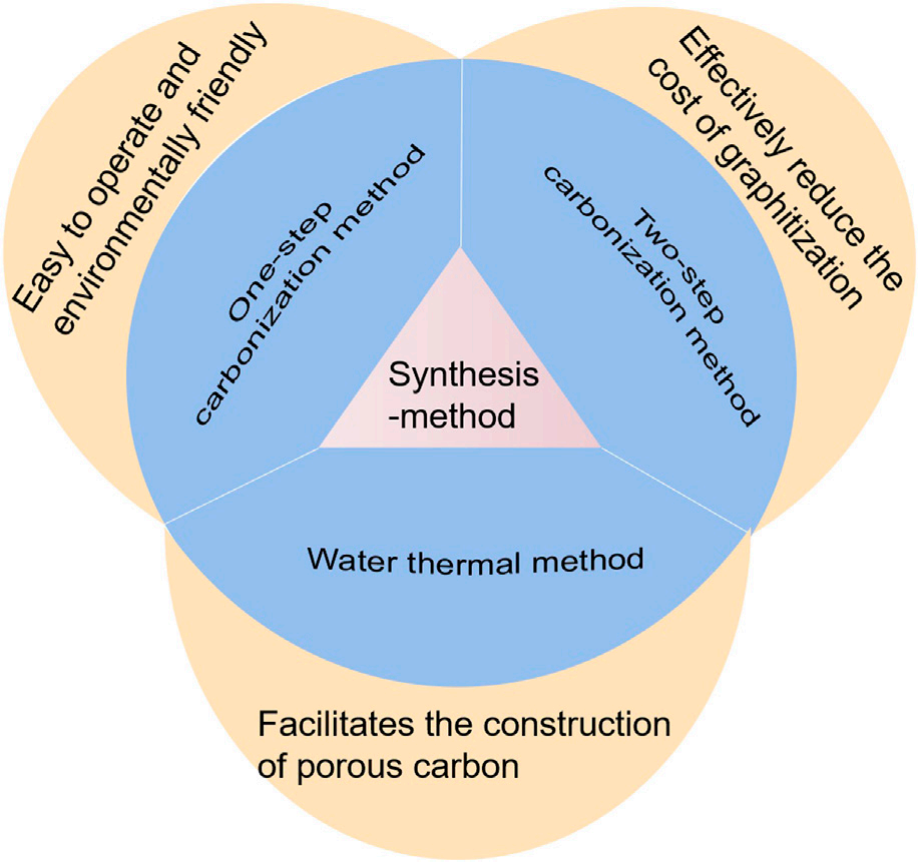
Different synthesis methods and characteristics.

The main synthesis methods of biomass carbon materials are one-step carbonization, two-step carbonization, and the hydrothermal method, respectively (Figure 1). Carbonization methods are usually accompanied by activation, pre-carbonization, acid–base treatment, ultrasound, and other treatments. After the material is pretreated and carbonized in the tube furnace, the structural properties of carbon materials are more stable. Hydrothermal methods are widely used. Medium- and low-temperature liquid phase controls are mainly used, and the process is simple. Without the use of high temperatures, a product with a complete crystal shape, uniform particle size distribution, and good dispersion can be obtained, resulting in lower energy consumption.

### 2.1 One-step carbonization method

Biomass precursors are typically immediately carbonized in a nitrogen or argon atmosphere in a one-step carbonization process. After pretreatment activation or ultrasonic treatment, it can also be carbonized. It is generally used to prepare pure biochar materials with advantages including simple operation and environmental protection (Alvira et al., 2022).

**FIGURE 2:**
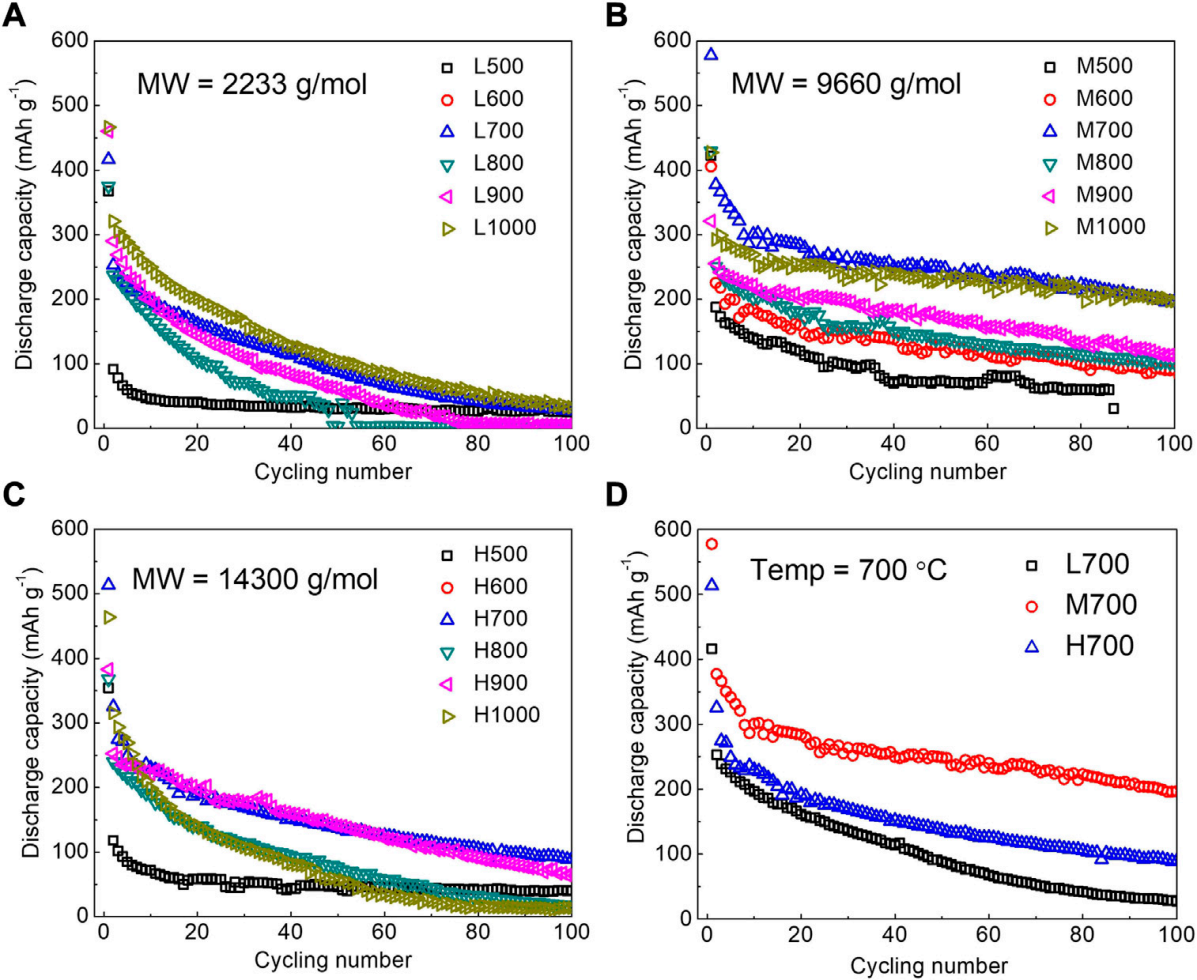
Cycling performance of L-HCS **(A)**, M-HCS **(B)**, and H-HCS **(C)** at pyrolysis temperatures ranging from 500°C to 1,000°C, L700, M700, and H700 **(D)**. All samples were measured at a current density of 50 mA·g^−1^ (Wu et al., 2022).

Wu et al. (2022) made use of lignin to carbonize directly without any pretreatment. Lignin is particularly important in cell wall formation, especially in wood and bark. Therefore, in plant biomass precursors, lignin content affects the structure and properties of carbon materials. In this study, the correlation among lignin molecular weight, pyrolysis temperature, and hard carbon structure was investigated by carbonization in a nitrogen atmosphere for 2 h at 500, 600, 700, 800, 900, and 1,000°C, respectively. High-molecular-weight lignin has relatively more pores at the same temperature. The higher the molecular weight of lignin is, the slower the conversion of lignin to disordered carbon will be. Among them, the carbon material formed by lignin carbonization at 700°C has the best performance such as a reversible specific capacity of 300 mAh·g^−1^, great rate of performance of 69% retention at 200 mAh·g^−1^, and good cycling stability of 79% retention after 100 cycles. In contrast, medium-molecular-weight lignin is superior to low-molecular-weight and high-molecular-weight lignin in terms of specific capacity and cyclic stability (Figure 2). Therefore, the molecular weight of lignin should be considered as a factor for the selection of plant biomass carbon material precursors.

**FIGURE 3:**
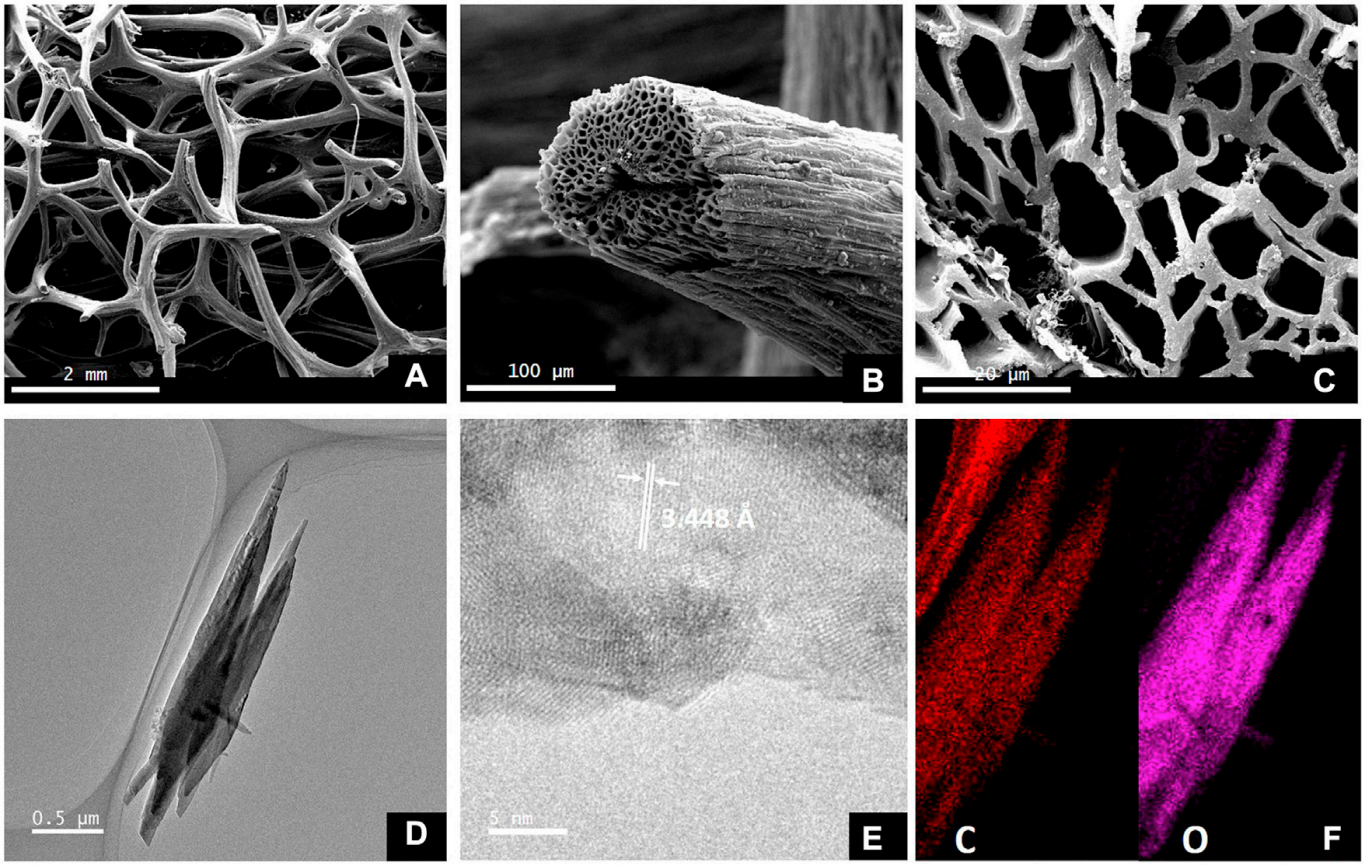
SEM **(A–C)** and TEM **(D–F)** of loofah-derived pseudo-graphite (LPG) (Wu et al., 2019).

In a single phase of carbonization, Wu et al. synthesized the loofah biomass carbon material (LPG) after first processing the material using alkali. The study reveals that alkali treatment may get rid of SiO_2_ and other impurities while producing a lot of active surface sites for storing potassium. The preparation of cellulose-based electrodes with high energy density, power density, and mass loading has received much attention recently because loofah is rich in cellulose. Loofah has a 3D interconnected multi-channel structure, which can be observed by transmission electron microscopy to have a graphite-like microcrystalline and amorphous “house of capillary” structure (Figure 3). In potassium-ion batteries, the specific reversible capacity is 150 mAh·g^−1^. At an LPG current density of 200 mAg^−1^, the reversible capacity is roughly 70 mAh·g^−1^. Due to cycle stability, after 400 cycles of 225 mAh·g^−1^, the Coulombic efficiency is near 100%.

The capacity for potassium storage is further impacted by the limitation of layer spacing and the restricted ability of acid–base therapy to boost the active site.

Experiments demonstrate that the synergistic effect of N doping and ZnCl_2_ molten salt can result in it having a pie-layered porous structure. Lu J F et al. mixed pinecone powder and ZnCl_2_ at different mass ratios and obtained pinecone-derived carbon using the molten-salt method combined with the one-step carbonization method. By adjusting the ZnCl_2_ addition ratio, it was discovered that with an increase in ZnCl_2_, the layer spacing, disorder degree, defect concentration, surface area, average pore diameter, and mesopore ratio of carbon materials increased first and then decreased. The total reversible capacity also increased first and then decreased. This structure has abundant defects and active sites as well as large layer spacing, which ensures the superiority and stability of potassium-ion battery performance (Jian-Fang et al., 2022).

### 2.2 Two-step carbonization method

**FIGURE 4:**
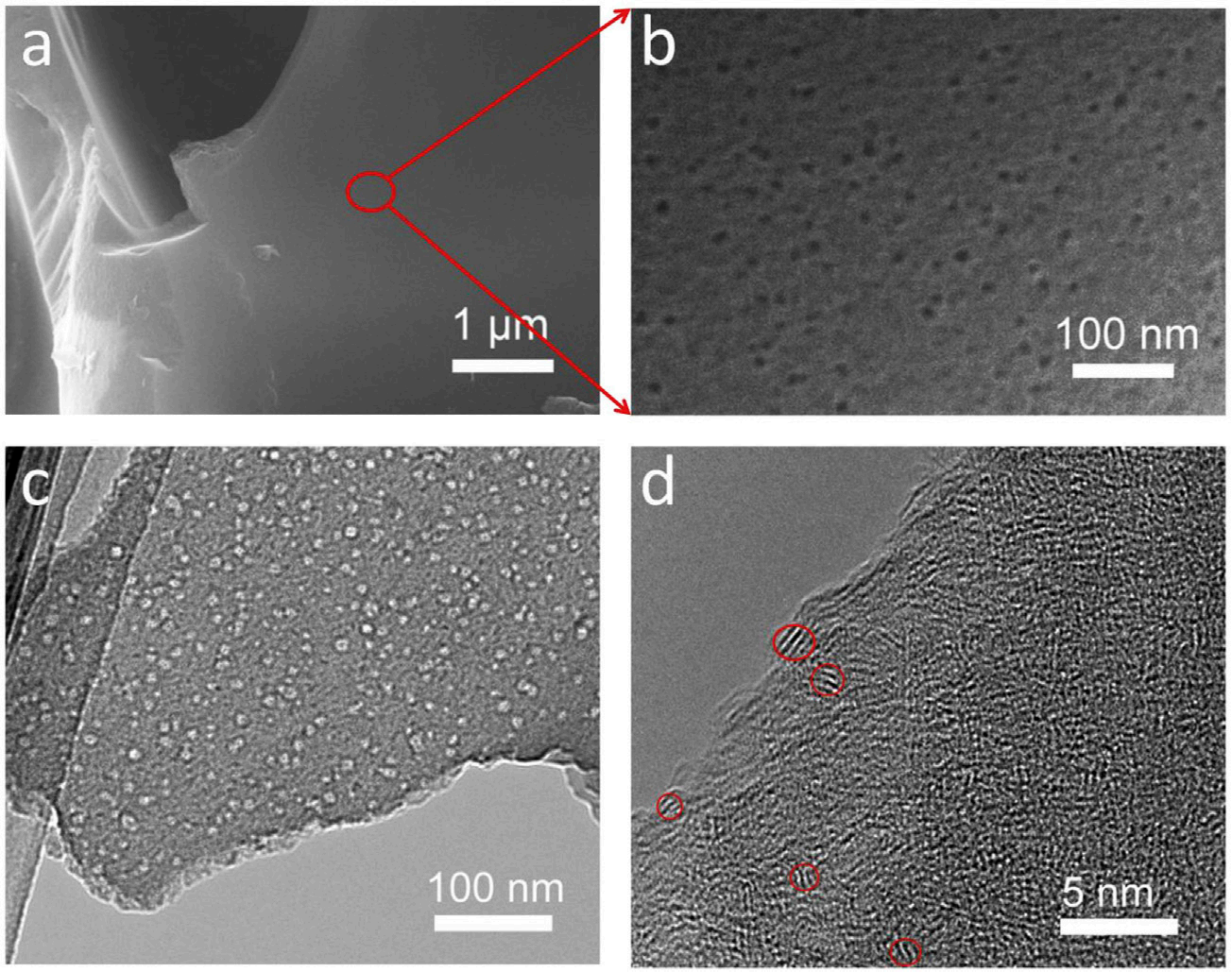
Representation of PBPC-1000: **(A, B)** SEM images, **(C)** TEM images, and **(D)** high-resolution TEM images (Cao, et al., 2018).

There are studies on the activation treatment of materials after primary carbonization, followed by secondary carbonization. Cao, et al. prepared potato biomass porous carbon (PBPC) materials with porous structure using the two-step carbonization method. Two-step carbonization typically requires the material to be pre-carbonized in a tube furnace first. Pre-carbonization can effectively reduce the cost of graphitization. SEM images (Figure 4) clearly show that there are many mesopores in the diameter range of 10 nm on the surface of PBPC, and the mesopores are continuous and smooth without any cracking. The potato precursor was pre-carbonized at 500°C for 3 h and then carbonized at 900, 1,000, and 1,100°C in an argon atmosphere. The microstructure of PBPC carbonized at various temperatures (PBPC-900, PBPC-1000, and PBPC-1100) was further identified by XRD patterns. After carbonization at 1,000°C, the material’s surface is uniformly covered in a large number of mesopores. Because of its porous nature, this material has more reaction and interaction sites, which promotes ion transport, and its disorder degree is lower than that of other samples. Through electrochemical performance tests, carbonization at a current density of 500 mA·g^−1^, and 400 cycles, the Coulombic efficiency is almost 100%.

The electrochemical properties of sucrose-based hard carbons (SHCs) and oak-based hard carbons (OHCs) were compared by Prabakar et al.’s (2017) team using a two-step carbonization process. It was discovered for the first time that hardwood (oak) can spontaneously generate interconnected channels with a diameter of micrometers to nanometers during carbonization at an optimized temperature (1,100°C). Because of this distinctive microstructure feature, the OHC1100 exhibits high resistance.

**FIGURE 5:**
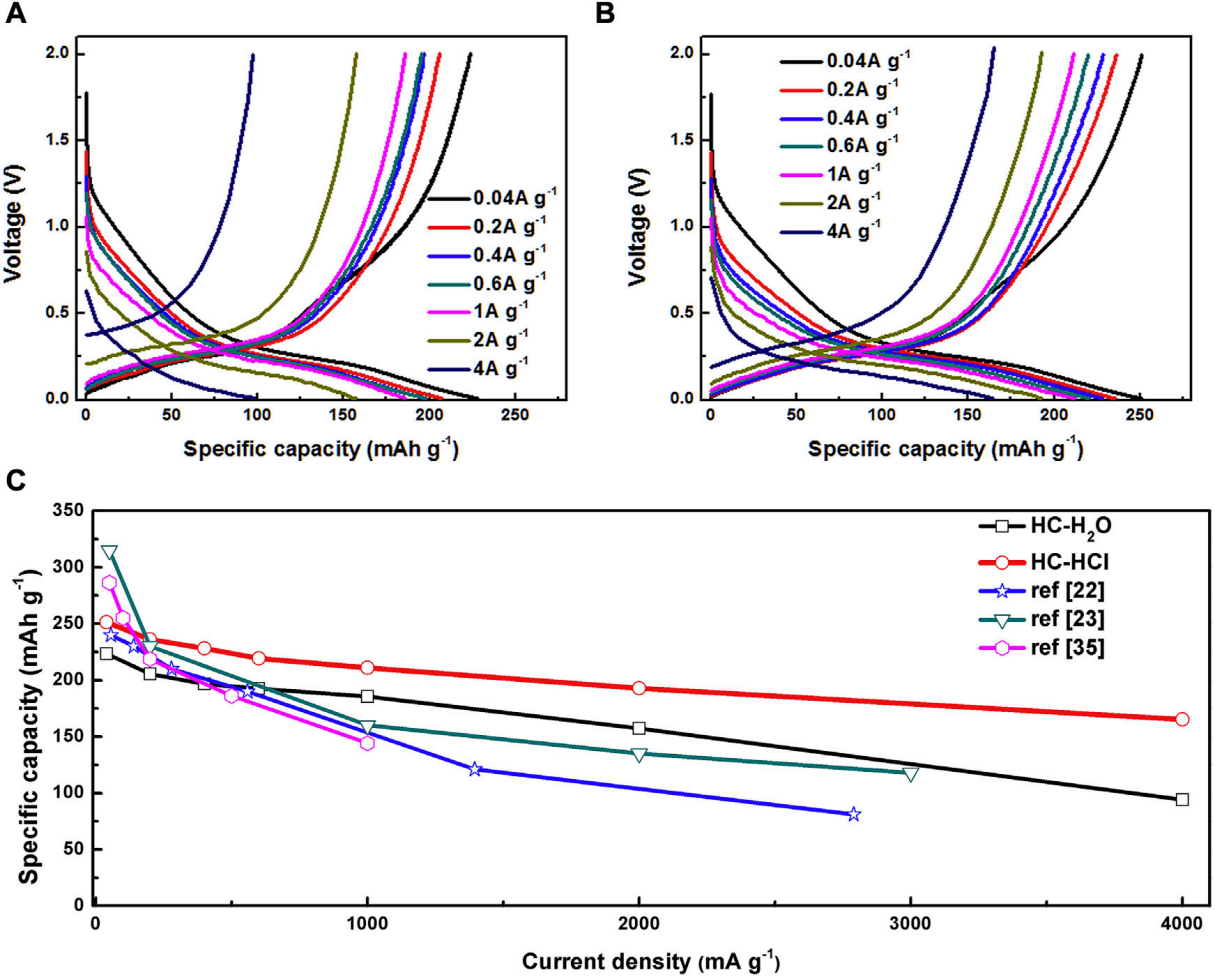
Magnification performance: **(A)** HC–H_2_O and **(B)** HC–HCL; **(C)** comparison with the literature (He, et al., 2018).

He et al. first treated defatted cotton with deionized water or HCL before decomposing it at 900°C for 2 h. Next, HC–H_2_O and HC–HCl were obtained by carbonizing at 1,200°C for 2 h in an argon atmosphere. Both carbonized samples showed hollow tubular structures and braided fiber morphologies. The highly polymerized carbon fiber chains are broken by acid treatment, which leads to an increase in the disorder of the initial structure. According to the electrochemical performance test (Figure 5), the reversible capacity of HC–HCL is 251.3, 236.1, 228.3, 219.4, or 211.1 mAh·g^−1^ when the current density is 0.04, 0.2, 0.4, 0.6, and 1 mAh·g^−1^, respectively. In comparison, the capacities of HC–H_2_O samples are 223.4, 205.6, 196.8, 192.6, and 185.7 mAh·g^−1^. Under the same current density, it is found that the rate performance of HC–HCL is superior to that of HC–H_2_O. Additionally, at high current densities of 2 and 4 Ag^−1^, 192.9 and 165.2 mAh·g^−1^, respectively, HC–HCLl displays especially high capacities. HC–H_2_O, on the other hand, can only supply 157.4 and 94.2 mAh·g^−1^, respectively.

While being a very straightforward process, one-step direct pyrolysis might not give the carbon atoms enough time to rearrange and subsequently generate a pseudo-graphite structure. Two-step carbonization, on the other hand, may result in a more pseudo-uniform hexagonal carbon plane embedded in a space of amorphous carbon. Consequently, the two-step strategy leads to the best ECI, reversible performance, and high velocity performance.

### 2.3 Hydrothermal method

**FIGURE 6:**
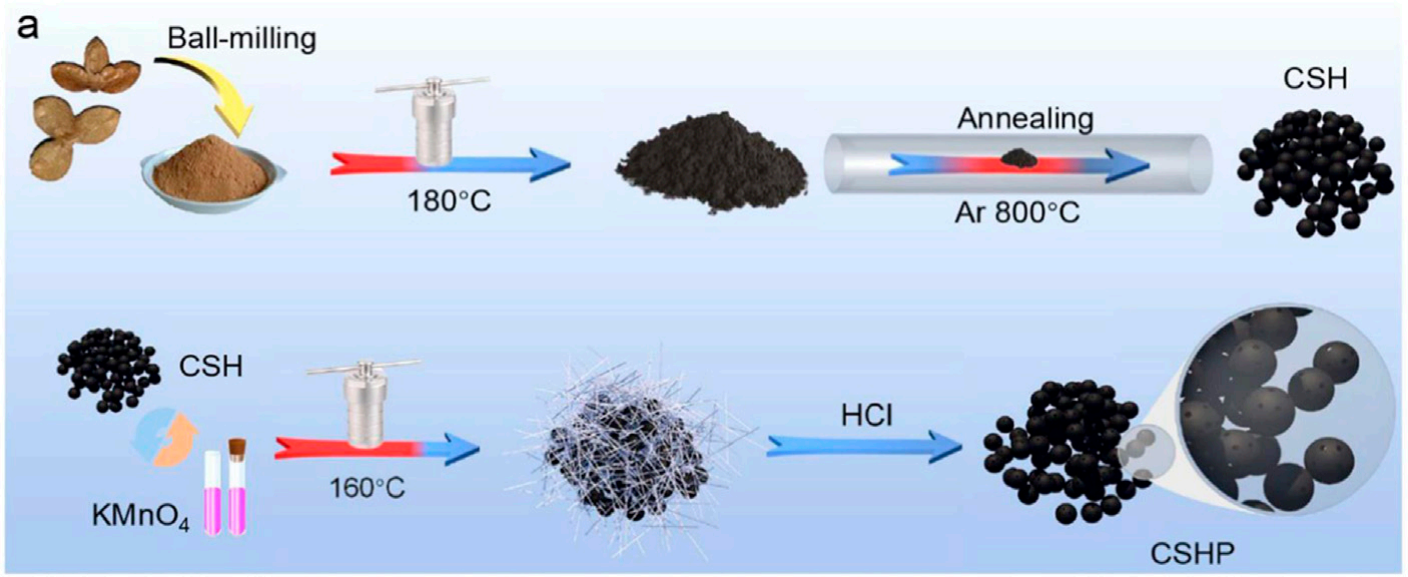
Schematic diagram of CSHP synthesis of porous carbon microspheres (Chen, et al., 2021).

The construction of biomass carbon materials can also be carried out using the hydrothermal method, which has the advantages of low cost and is not easy to cause pollution. However, the reaction needs to be carried out in a specific reaction vessel, namely, a reaction kettle. Chen et al. constructed porous carbon microspheres (CSHP) directly from the raw waste biomass of the camellia shell by a simple two-step hydrothermal method. The processed camellia shell powder was dispersed in deionized water, heated at 180°C for 24 h, and then, annealed at 800°C for 2 h under an Ar atmosphere to complete a primary hydrothermal carbonization. After secondary hydrothermal treatment with potassium permanganate, the surface of the carbon sphere becomes rougher and has more micropores, which facilitates the storage of potassium ions (Figure 6). After 100 cycles at 100 mA·g^−1^, the specific capacity of 237.6 mA·hg^−1^ is still provided, and the capacity retention rate is 89.8%. It has a specific capacity of 264.5 mAh g^−1^ at a current density of 100 mA g^−1^, and its microporous structure and spherical morphology make it have good electrochemical performance.

Pu Q et al. prepared carbon materials from chestnut shell (CS), walnut shell (WS), and macadamia nut shell (MS) using the hydrothermal method. The raw materials were first hydrothermally reacted with deionized water at 250°C for 2 h, mixed with KOH, and then, placed on a magnetic stirrer for 12 h. The dried samples were placed in a tube furnace for carbonization (Qiuyun et al., 2021). Chen C utilized glucose to synthesize hard carbon microspheres by the hydrothermal reaction. First, glucose was dispersed in deionized water and heated at 200°C for 2 h, then the dried powder was mixed with melamine in an argon atmosphere, and annealed at 900°C for 5 h to obtain a uniform spherical hard carbon with a diameter of about 3 μm. Particle composition of N-SHC: In potassium-ion batteries, N-SHC provides a high reversible capacity of 248 mAh·g^−1^. The rate of performance is 251, 206, 152, and 93 mAh·g^−1^ at current densities of 200, 500, 1,000, and 2000 mA·g^−1^, respectively. In addition, the nitrogen-doped N-SHC material also exhibits excellent long-term cycling stability, in which the N-SHC electrode maintains a high reversible capacity at 200 mAh·g^−1^ with a capacity retention rate of 81% after 600 cycles (Chen et al., 2022).

The preparation of biomass carbon materials is relatively mature at present, and the direct one-step carbonization method mostly yields a pure biomass carbon. The treatment method can yield carbon materials with excellent structure, which can be used for negative electrode research of potassium-ion batteries, sodium-ion batteries, lithium-ion batteries, etc. (Ahmed et al., 2021).

## 3 The effect of single-atom doping on the modification of biomass carbon materials

The preparation method of pure carbon biomass carbon materials and the structure is relatively simple, and the unique advantages of biomass carbon materials cannot be exerted. Although hard carbon materials have great potential in potassium-ion batteries, pure carbon materials mainly use an intercalation mechanism during potassium storage. Its limited potassium storage capacity and slow reaction kinetics result in low-energy density and rate capability of pure carbon materials. Therefore, atomic doping of pure carbon materials is required. Heteroatom doping can make a large number of active sites appear on the surface of the material, which is conducive to the storage of alkali-metal ions. It can also improve the transmission speed of ions/electrons to improve the rate capability of carbon materials (Kim et al., 2021). Doping is generally divided into single-atom doping and polyatomic doping. Common doping atoms are mainly N, S, P, and F (Yang et al., 2021).

### 3.1 N-doped carbon

The nitrogen atoms are more widely doped, including pyrrolic N, pyridinic N, graphitic/quaternary N, and oxidized N. In order to increase the surface hydrophilicity of electrode materials, nitrogen functional groups, which are hydrophilic functional groups, can facilitate the contact between electrolyte ions and electrodes. Furthermore, it can enhance the electrochemical performance of biomass carbon materials by increasing their void structure.

**FIGURE 7:**
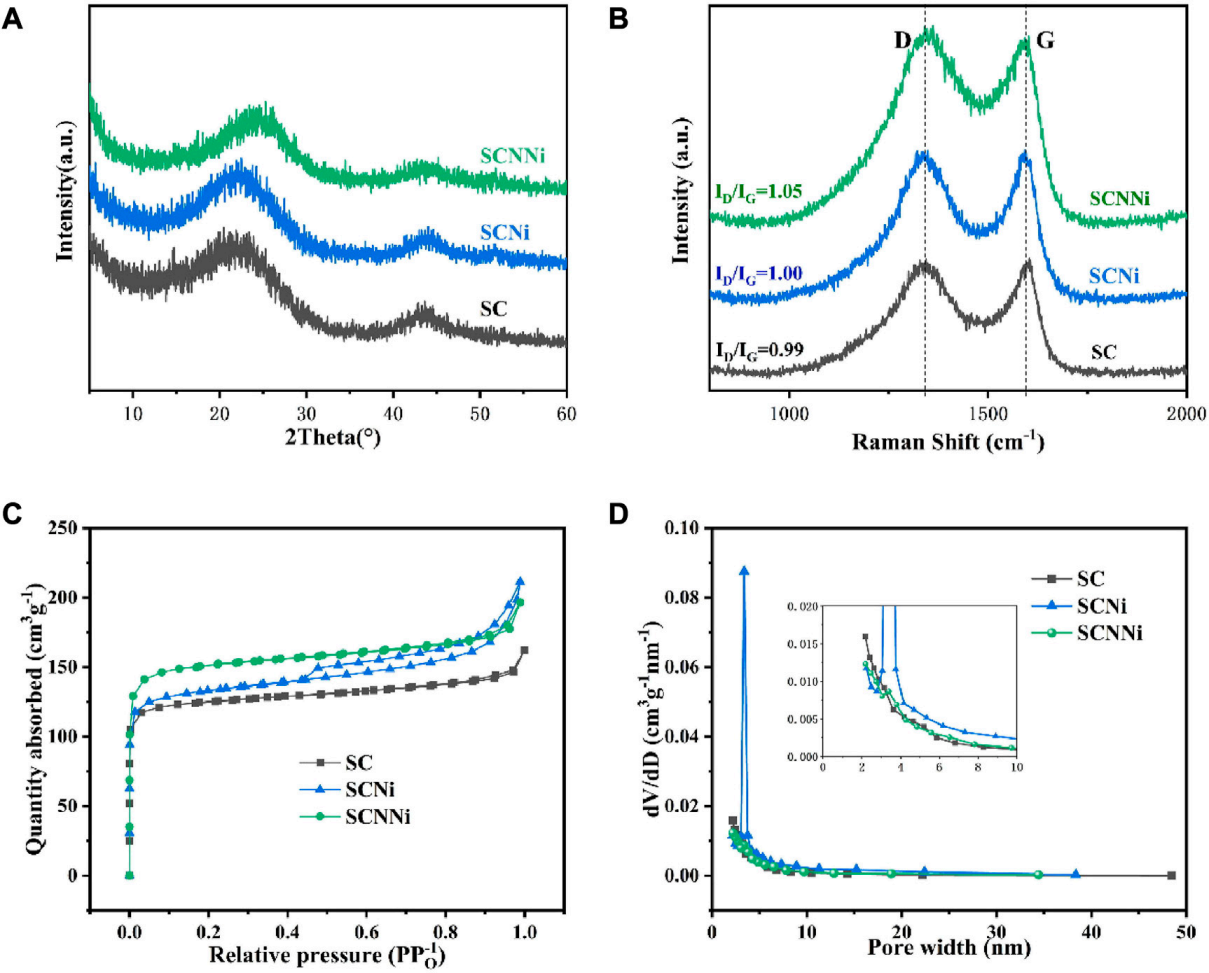
**(A)** XRD patterns and **(B)** Raman spectra of SC, SCNi, and SCNNi. **(C)** Nitrogen adsorption–desorption isotherms. **(D)** Pore size distributions of SC, SCNi, and SCNNi (Qijiu, et al., 2021).

Deng Q’s team used bagasse as a raw material to prepare an N-doped three-dimensional porous carbon material as a research material on the electrochemical performance of the potassium-ion battery anode material. After activation with KOH, it is mixed with urea and NiCl_2_ in a certain proportion. Then, it was annealed at 800°C for 2 h under a N_2_ atmosphere to prepare SCNNi, SCNi, and SC materials. Nitrogen-doped and Ni-porous SCNNi samples had the lowest degree of graphitization. Due to the increase in oxygen/nitrogen functional groups after nitrogen doping and severe decomposition under high-temperature pyrolysis, the defects increase and the ordering weakens. Compared with the other two samples, the synthesized SCNNi sample has a larger specific surface area, superior average pore size, and specific pore volume, which provides a channel for K+ transport and enhanced potassium-storage capacity. It is more favorable for the insertion and extraction of K ions in the host material (Figure 7). After 50 cycles, the SCNNi sample exhibits a reversible specific capacity of 165.2 mAh·g^−1^ at a current density of 100 mA·g^−1^. The specific capacity of the SCNNi sample is 100.4 mAh·g^−1^ after 400 cycles at a current density of 200 mA·g^−1^ (Qijiu et al., 2021).

Wang X’s team ground dandelion seeds (DS) into a powder and mixed them with an activator (KOH) at a ratio of 1:1 in water and then annealed at 800°C for 2 h in an argon atmosphere. After washing with HCL again, a porous carbon material was prepared. After activation, the specific capacity increased from 168 mAh·g^−1^ to 255 mAh·g^−1^. At the same time, capacity retention was also increased from 87% to 91% (Wang J et al., 2020).

Luo H added silk into the mixed solution of ZnCl_2_ and FeCl_3_. After treatment, it was rapidly heated at 150°C for 1 h at a heating rate of 5°C min^−1^ to remove moisture. SHPNC samples were then prepared by carbonization at 750°C, 900°C, and 1,050°C for 1 h. From the voltammetry curves of the three samples, it is shown that SHPNC-900 has a larger peak area and higher specific capacity. SHPNC-900 delivers a high reversible capacity of 300 mAh·g^−1^ at a current density of 25 mA·g^−1^ after 163 cycles. At a current density of 200 mA·g^−1^, the SHPNC-900 electrode showed a high reversible capacity of 270 mA·g^−1^ after 923 cycles. Stable cycling performance showed high specific capacities of 271 (50 mA·g^−1^) and 164 mAh·g^−1^ (1,000 mA·g^−1^) after 330 and 593 cycles, respectively (Luo et al., 2020).

### 3.2 P-doped carbon

Nitrogen and phosphorus atoms share the same valence electron. P–C and P–O bonds can be used to dope it into the carbon layer. Yet, because of its larger radius and ability to donate electrons, the P atom can both widen the layer spacing and significantly boost the electrical conductivity of hard carbon materials.

Alvin et al. found that although heteroatom doping can cause a large number of defect sites, excess defect sites can cause a lower initial Coulombic efficiency (ICE). An experimental approach combining high-temperature carbonization and low levels of heteroatom doping can improve this situation. This study uses lignin as a precursor for P doping. The purified lignin was mixed with phosphoric acid to filter out unabsorbed PO_4_^3-^ to avoid excessive doping of P and prevent hard carbon activation and then carbonized at 1,300°C. After P doping, the low-voltage capacity below 0.25 V increases from 153 mAh·g^−1^ to 192 mAh·g^−1^, and the total reversible capacity increases from 245 mAh·g^−1^ to 302 mAh·g^−1^. This experiment led to further research on the amount of heteroatom doping and defect sites in future experiments. Experimental results demonstrate that low-level P doping of lignin-derived hard carbons is an efficient approach to develop high-energy-density anodes with the high ICE.

Yong Liu’s team mixed flour with a phosphoric acid solution to form a homogeneous gel-like substance. The samples were dried under an argon atmosphere at a heating rate of 3°C min^−1^ and annealed at 600°C, 700°C, and 800°C for 3 h, respectively. Finally, PPDC was prepared by washing with HCL. Carbon materials were prepared from cheap flour as carbon source have the characteristics of high-specific surface area and hierarchical porous structure. The P atom doping can provide more active sites for K+ storage and enhance its conductivity. It is found that the elements of C, O, and P are uniformly distributed in the carbon skeleton through SEM images. The difference is that the element content varies with temperature. Too high and too low temperatures are not conducive to PPDC as an excellent anode material for potassium-ion batteries. When the annealing temperature is 700°C, both the pore structure and the electrochemical performance are the best. The initial discharge capacity of PPDC-700 is 565 mAh·g^−1^, and the charge capacity is 376 mAh·g^−1^. After 50 consecutive cycles, the reversible capacity of PPDC-700 is 310 mAh·g^−1^, indicating that PPDC-700 has high reversible capacity and good cycling stability (Yong et al., 2022).

### 3.3 S-doped carbon

Sulfur atoms can increase the interlayer distance of carbon. Doping involves replacing carbon atoms, which causes more vacancies and flaws overall. This allows for more electrolyte ions to be accommodated while charging and discharging. Its oxygen reduction activity can be exploited as an active site in the generated C–S–C bond, enhancing its electrocatalytic activity and cycling stability. The interlayer spacing of S-doped hard carbon is the same as that of pure hard carbon and sometimes even smaller. The bond length of the C–S bond (1.78 Å) is about 16% longer than that of the C–C bond (1.53 Å), resulting in lattice gap stretching, which is beneficial for ion storage. And sulfur doping can also increase the hydrophilicity and conductivity of carbon materials (Ma et al., 2022).

Tian et al. first used KOH to activate the treated bamboo powder. The main effect of activation is to expand the specific surface area. It was then hydrothermally treated at 150°C for 12 h. After annealing at 700°C for 2 h, the material was vulcanized with sulfur powder of different qualities to finally obtain S-BC. The study found that with the increase in the sulfur doping content, the specific surface area of the material did not change much. However, the surface microstructure of materials prepared with different carbon–sulfur ratios was different. With the increase in the sulfur content, the proportion of sulfur atoms in the material increased and the oxygen content also increased. This phenomenon will lead to the as-synthesized S-BC materials exhibiting different electrochemical performances, and the enhanced electrochemical performance is mainly attributed to the C-S-K bond, which has a higher K-ion storage capacity than carbon (KC8). The material exhibits good chemical properties, and S-BC^−1^ provides a maximum specific capacity of 296.6 mAhg^−1^ at a current density of 50 mA·g^−1^ after 50 cycles. Even at a high current density of 200 A·g^−1^, the specific capacity of 203.8 mAh·g^−1^ was maintained after 300 cycles. The excellent performance of S-BC is attributed to the activation of KOH and to the synergistic effect of moderate doping of sulfur.

**FIGURE 8:**
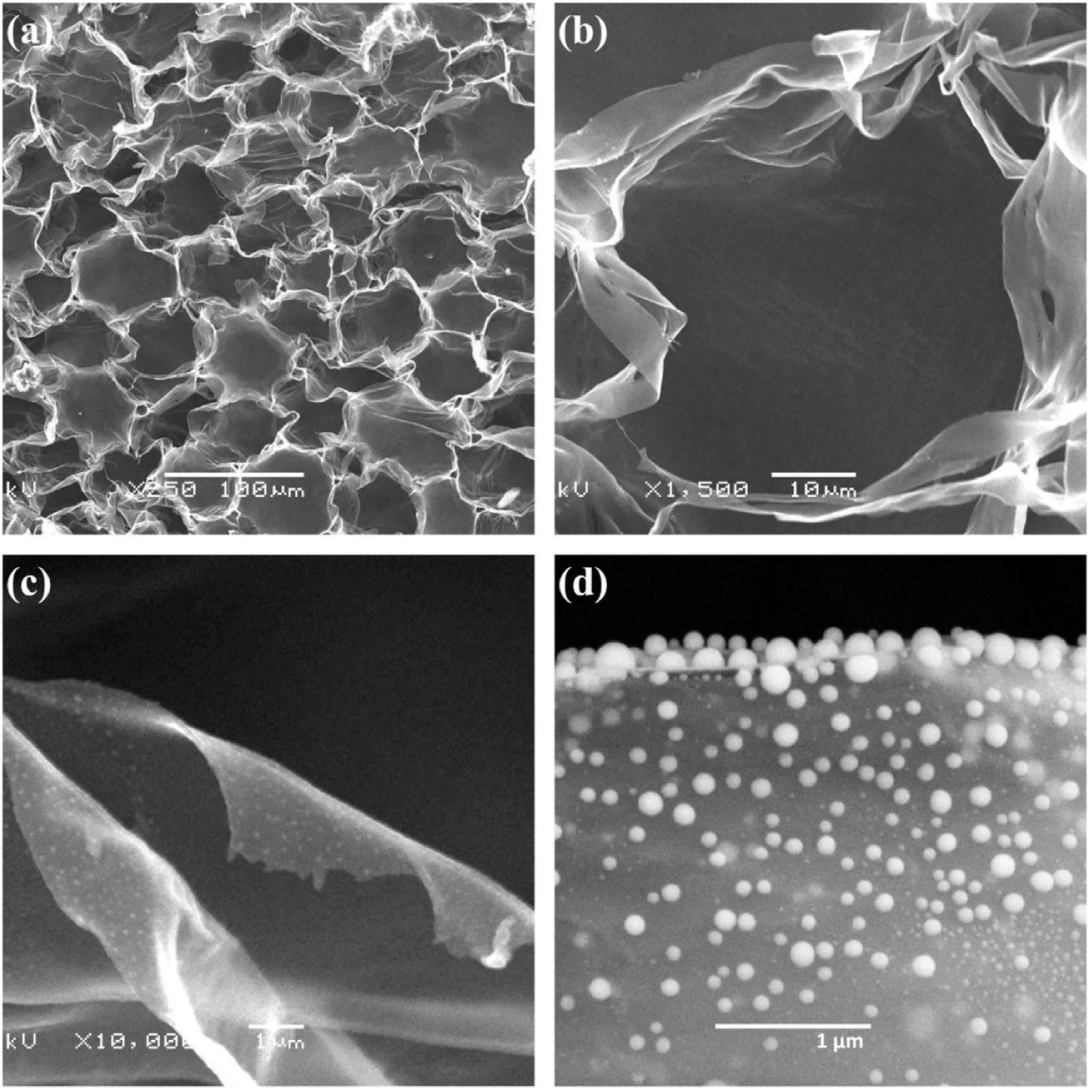
**(A–D)** SEM images of CHP/S at different magnifications (Wang, et al., 2021).

Wang P and other teams took out the hemp core to dry and continuously dripped the sulfur solution on the hemp core. It was then heated to 800°C in argon for 3 h to obtain CHP/S. CHP/S has a coiled structure due to the infiltration of sulfur melting (Figure 8). Because the increase in temperature favors the fast transport of ions, the team investigated the rate capability of the CHP/S electrode at different temperatures. At 60°C, the discharge capacity of the electrode is 589 mAh·g^−1^ when the current density is 30 mA·g^−1^. When the current density is 2000 mA·g^−1^, the discharge capacity is 260 mAh·g^−1^. In addition, the excellent rate performance of the electrode at 60°C far exceeds that at 25°C (Wang et al., 2021).

Zhang Y’s team prepared high-sulfur-doped hard carbon with advanced potassium-storage ability using the molten salt method. Glucose as the carbon source and potassium sulfate as the sulfur source were mixed and thoroughly ground, mixed with potassium chloride (KCl, 2.2 g) and lithium chloride (LiCl, 1.8 g), and then, annealed at 700°C in an argon atmosphere for continuous 5 h. Finally, it was washed and dried with hydrochloric acid to obtain HS–HC. The surface of HS–HC is rough and porous, and the carbon blocks are composed of different shapes. The porosity of carbon blocks can be attributed to the reaction of carbon intermediates with K_2_SO_4_. The electrochemical performance is good due to the reaction of sulfur and potassium, providing a high capacity of 361.4 mAh·g^−1^ on initial cycling. It also provides a high capacity of 317.7 mAh·g^−1^ at the 100th cycle at a current density of 0.05 A·g^−1^, while the capacity is low at lower sulfur content (Zhang et al., 2020).

### 3.4 Other atomic doping

Similarly, Wang et al. (2021) synthesized biochar doped with three different fluorinating agents using the hemp straw as a precursor. Biomass carbon (PTFE-CHEMP) treated with polytetrafluoroethylene PTFE contains the most defects due to being wrapped by fluorine-containing nanotubes and has the largest amount of F doping and the best pore size. It has excellent electrochemical performance as an anode material for potassium-ion batteries and can still provide an average reversible capacity of 369.6 mAh·g^−1^ after 500 cycles at a current density of 200 mA·g^−1^. Having excellent rate performance, even at a high current of 2000 mA·g^−1^, it can still provide a specific capacity of 229.3 mAh·g^−1^. Fluorine has a strong electronegativity, which increases layer spacing while also lowering the energy barrier for embedding potassium ions.

Although the electrochemical performance of single-atom doping has been improved compared with pure biomass carbon materials, there are still some deficiencies in improving specific capacity, ICE, rate performance, and long-cycle stability. The study found that the synergistic effect of co-doping of multiple heteroatoms can more effectively improve the potassium-storage performance of carbon materials.

## 4 The effect of multi-heteroatom doping on the modification of biomass carbon materials

At present, the most common atomic doping are N/O double doping, N/P double doping, N/S double doping, etc., and some experiments have also conducted research on polyatomic doping of biomass carbon materials (Wang X et al., 2020).

**FIGURE 9:**
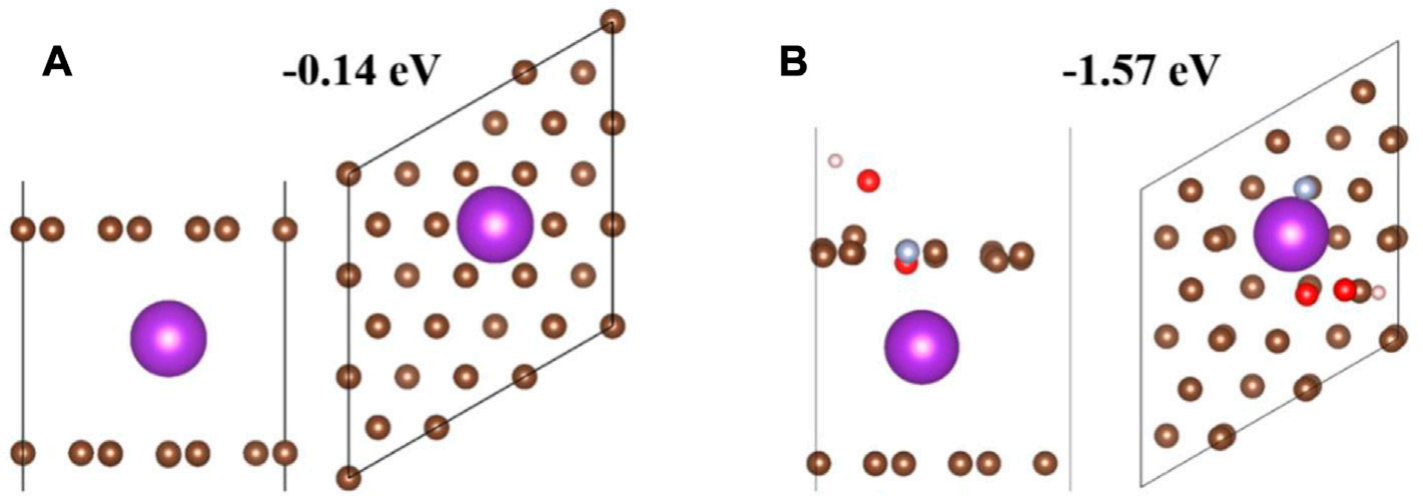
Theoretical simulation of K adsorption in different structures. Side and top views of single K atoms adsorbed on **(A)** hard carbon (HC) and **(B)** O/OH/pyrrole N co-doped HC. The purple balls represent potassium atoms, the gray-blue balls represent nitrogen atoms, the brown balls represent carbon atoms, the red balls represent oxygen atoms, and the white balls represent hydrogen atoms (Yang, et al., 2021).

*Ganoderma lucidum* spore powder is egg shaped under the microscope, and its structure is similar to that of pecans, with a porous cage-like structure. Yang et al. annealed *Ganoderma lucidum* spore powder at 850°C for 3 h in nitrogen and then dispersed in a mixed solution of distilled water and diethylenetriamine. After ultrasonic treatment, a certain amount of ammonium molybdate tetrahydrate was added and then ammonium molybdate tetrahydrate introduced more O. After a series of treatments, it was treated at 850°C for 2 h in an N_2_ atmosphere. SEM images observed that the cage-like structure was still maintained, which ensured excellent electrical conductivity. O/OH/pyrrole N co-doping had larger specific surface area and adsorption energy than directly annealed carbon materials. The contact area of the electrolyte is conducive to the capture and storage of potassium ions, shortening the ion migration path, and can also improve the K+ storage capacity and K+ insertion/de-insertion speed (Figure 9). This indicates that N/O co-doping, the coexistence of hydroxyl (OH) and O/pyrrole N, further enhances the K adsorption capacity and improves the cycling performance. In summary, the *Ganoderma lucidum* spore powder biomass carbon material has good electrochemical performance and can provide a reversible specific capacity of 251.2 mAh·g^−1^ after 1,500 cycles at a current density of 0.5 A·g^−1^. It also provides a reversible specific capacity of 334.6 mAh·g^−1^ after 2000 cycles at a current density of 5 A·g^−1^. The reversible specific capacity of 124.19 mAh·g^−1^ can still be maintained after 5,000 cycles at a current density of 10 A·g^−1^.

In addition to N/O doping, N/S double doping has also been performed on biochar. Wan et al. (2021) pretreated the drug residues, activated them with KOH, added melamine and thioacetamide in a certain proportion, and then, heated them under argon at 800°C for 2 h. The material exhibits a disordered network porous structure, and the resulting edge defects and pore structure can improve electrical conductivity and provide sufficient active sites.

Xu B’s team studied the O/S co-doping of biomass carbon. They used the absorbent cotton to prepare O/S co-doped H–OS–C materials through a simple hydrolysis–vulcanization process. Raman spectroscopy shows that the carbonized material after hydrolysis has more defect sites than the directly carbonized material because hydrolysis brings more oxygen groups (-OH and -COOH). H–OS–C delivers high capacities of 409, 322, 255, and 185 mAh·g^−1^ at current densities of 0.1, 0.2, 0.5, and 1 A·g^−1^, respectively. Excellent rate performance maintains a reversible capacity of 135 mAh·g^−1^ at a high current density of 2 A·g^−1^ (Xu et al., 2020).

**FIGURE 10:**
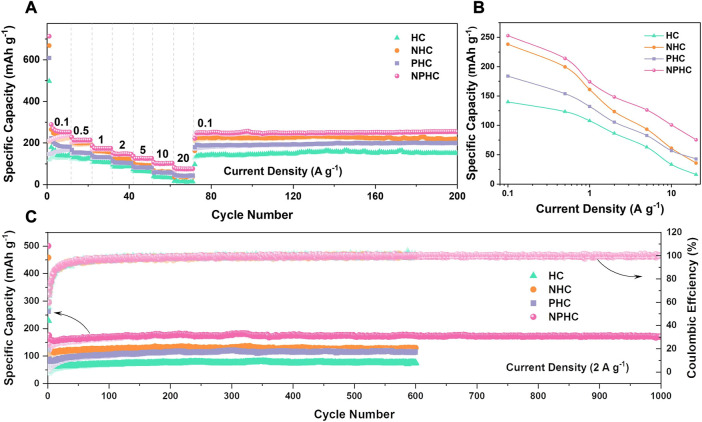
Comparison of potassium storage capacity of HC, NHC, PHC and NPHC. **(A)** Rate capability **(B)** Specific capacity at different current densities **(C)** Cycle life of 2 A g^-1^ (Wang, et al., 2022).

Wang et al. (2022a) used cellulose to prepare N/P co-doped biomass carbon materials to study the capacity and rate performance of potassium-ion batteries. Graphite interlayer spacing measurements show that the effect of P doping on the increase in interlayer spacing is more pronounced than that of N doping, and N/P co-doping can further improve the interlayer spacing of pristine hard carbon. N doping alone improves the initial capacity of hard carbon and potassium-storage capacity at low current densities, and the improvement effect of P doping is comprehensive. A synergistic effect can be exerted when N/P co-doped, in which the N/P co-doped biomass carbon structure is superior to the corresponding single-heteroatom-doped graphitic carbon structure for potassium affinity and electronic conductivity. At a high current density of 2 A·g^−1^, the N/P co-doped carbon exhibited a specific capacity of 172 mAh·g^−1^ after 600 cycles (Figure 10). It is shown that the N/P co-doped hard carbon exhibits higher potassium-storage capacity and excellent cycling stability compared to the pristine hard carbon.

As part of their research into N/P doping, Yuan X’s team created 3D-HPCS anode materials using chicken bones as the raw carbon source. This substance, as opposed to cellulosic biomass carbon, has unusual pores that resemble forests and offer plenty of open holes for effective ion transport and electrolyte penetration. At a current density of 0.2°C, the 3D-HPCS anode has a maximum specific capacity of 205 mAh·g^−1^ after 450 cycles. Even at a high current density of 2.0°C, a specific capacity of 113 mAh·g^−1^ can be attained (Yuan et al., 2021).

It is not difficult to see that the performance of diatomic-doped biomass carbon is significantly better than pure biomass carbon and single-atom-doped biomass carbon. Polyatomic doping can have a synergistic effect. This effect can provide more active sites and functional groups. It also promotes the surface wettability of the material and improves the electrochemical properties compared to single doping (Ruan et al., 2019; Tao et al., 2021; Gao et al., 2022). In addition to diatomic doping, some people have studied multi-heteroatom doping. This doping method is relatively complex, but its battery performance is significantly better than diatomic doping.

A species of fungus called puffball is used in traditional Chinese medicine. The puffball’s top is a thin, pliable sealing lid that keeps the spores inside from contact with the outside world and safeguards them until they reach maturity. Yang et al. (2021a) created nitrogen, oxygen, and phosphorus ternary-doped hollow biomass carbon spheres (NOP-PBs) with a sponge-like inner cap after removing the outer cap. The natural hollow spherical structure of puffball spore powder is carried over into the NOP-PB substance. The transport and diffusion of electrolyte ions inside the material are not aided by the simple spherical structure, but this special inner cavity structure can significantly reduce stress and strain brought on by the insertion and removal of potassium ions as well as changes in the electrode material’s structure and volume. At 500 mA·g^−1^, the cycling capabilities of NOP-PB and PB electrodes were examined. The results show that the material (NOP-PB-2) electrode with the addition of 3 mL of tributyl phosphate has the highest reversible capacity and long-cycle stability, and it provides a reversible capacity of 408.9 mAh·g^−1^ after 100 cycles. Until the end of the cycle (600 cycles), it can still maintain a high reversible capacity of 352.22 mAh·g^−1^, and the Coulombic efficiency remains around 97.5%. At the same time, the cycle performance of NOP-PB electrode is much higher than that of the PB electrode, indicating that the artificial adjustment of doping type (N, O, and P ternary co-doping) and doping amount can effectively improve the cycling performance of the electrode.

In order to better compare different modification strategies, we summarized the electrochemical performance of various types of carbon-based materials for the anode of PIBs in Table 1.

**TABLE 1:** Potassium-storage properties of the carbon-based anode materials were compared for different synthesis methods and modification strategies.

	Material	Current density (mA·g^−1^)	Cycle number	Specific capacity (mAh·g^−1^)	Reference
One-step carbonization method	Lignin	300	100	200.0	Wu, et al. (2022)
	Loofah biomass carbon material	200	400	225.0	Wu, et al. (2019)
Two-step carbonization method	Potato carbonization material	500	400	196.0	Cao, et al. (2018)
	Defatted cotton	40	150	253.0	Jian-Fang, et al. (2022)
Hydrothermal method	Camellia shell	100	100	237.6.0	Chen, et al. (2021)
	Glucose	200	600	248.0	Chen, et al. (2022)
N-doped carbon	Bagasse	100	50	165.2	Qijiu, et al. (2021)
	Dandelion seeds	50	100	170.52	Wang J et al. (2020)
	Silk	200	923	270.0	Luo, et al. (2020)
P-doped carbon	Lignin	300	700	0.02	Alvin, et al. (2019)
	Flour	100	80	292.0	Yong, et al. (2022)
S-doped carbon	Bamboo powder	50	50	296.6	Tian, et al. (2019)
	Hemp core	30	60	591.7	Wang, et al. (2021)
	Glucose	50	100	317.7	Zhang, et al. (2020)
Multi-heteroatom doping	*Ganoderma lucidum* spore powder	500	1,500	251.2	Yang, et al. (2021)
	Absorbent cotton	2000	500	120.0	Xu, et al. (2020)
	Cellulose	2000	600	172.0	Wang, et al. (2022)
	Chicken bones	54.6	450	205.0	Tao, et al. (2021)
	Puffball	500	100	408.9	Yang, et al. (2021)

## 5 Conclusion

Focusing on the development of biocarbon material research over the past few years, numerous biomass materials have shown to be excellent candidates for use as energy storage materials. As materials for potassium-ion batteries, they also display good electrochemical performance, opening up a new important area for the use of novel environmentally acceptable materials. The application of atomically doped biomass carbon materials as anode materials for potassium-ion batteries and recent advancements in the fabrication of pure biomass carbon materials with various shapes and sizes are both covered in this work. While developing biomass carbon materials, the following issues need still be taken into consideration: 1) the simpler production procedure and comparatively reduced production costs of biomass carbon composites over synthetic materials are two of its key advantages. Furthermore, waste resources can be used to achieve mass manufacturing, lessen resource waste, and get the product closer to commercialization. 2) The advantages of biomass carbon materials should be completely utilized, and the use of expensive and/or dangerous chemicals should be avoided, in order to achieve environmental protection. It is encouraged to synthesize carbon compounds at mild temperatures. 3) ICE development: Low ICE is brought on by both an irreversible potassium buildup in pores, functional groups, and interlayer gaps and a certain electrolyte breakdown. Chemical pretreatment and a reduction in the number of additives can improve ICE. Moreover, future research will focus on finding high-performance negative electrode materials with improved electrochemical performance.
